# Investigating the risks of late preterm and term neonatal morbidity across clinical subtypes of intrahepatic cholestasis of pregnancy

**DOI:** 10.3389/fmed.2025.1528705

**Published:** 2025-03-14

**Authors:** Wei-Zhen Tang, Yi-Fan Zhao, Lan Wang, Qin-Yu Cai, Wei-Ze Xu, Li Wen, Xue-Bing Chen, Ting-He Sheng, Tian-Qi Fan, Tai-Hang Liu, Rong Li, Shang-Jing Liu

**Affiliations:** ^1^Department of Bioinformatics, School of Basic Medical Sciences, Chongqing Medical University, Chongqing, China; ^2^Department of Obstetrics and Gynecology, Women and Children’s Hospital of Chongqing Medical University, Chongqing, China; ^3^Department of Obstetrics, The First Affiliated Hospital of Chongqing Medical University, Chongqing, China

**Keywords:** intrahepatic cholestasis of pregnancy, pregnancy outcome, subtype of ICP, neonatal intensive care unit, ursodeoxycholic acid

## Abstract

**Background:**

This investigation assesses the perinatal risks associated with different clinical subtypes of intrahepatic cholestasis of pregnancy (ICP) based on clinical symptomatology, with the goal of informing optimal delivery timing for each specific ICP subtype.

**Study design:**

The retrospective study encompassed 2,057 singleton pregnancies with ICP, categorized into the single-symptomatic (ICP-S) and the multisymptomatic (ICP-M) groups. The ICP-M group was further subdivided based on symptom combinations: elevated TBA with elevated transaminases (ICP-M_T_), elevated TBA with pruritus (ICP-M_P_), and combined elevations with pruritus (ICP-M_B_). The investigation included an assessment of baseline characteristics, a comparison of perinatal outcomes between the ICP-S and ICP-M groups, an evaluation of the impact of ursodeoxycholic acid and second-line treatments, and the analysis of severe adverse neonatal outcomes by clinical classification and gestational age through the logistic regression and restricted cubic spline methods.

**Results:**

Baseline characteristics suggested *in vitro* fertilization (IVF) and nullipara as more prevalent in the ICP-M, which also had an earlier diagnosis of ICP than in the ICP-S. In addition, the ICP-M exhibited higher liver function and blood glucose levels. The ICP-M was significantly associated with increased risks of gestational diabetes mellitus (GDM) (OR 1.57), preterm birth (OR 1.92), low-birth-weight infant (OR 1.81), and neonatal intensive care unit (NICU) admissions (OR 1.48) than the ICP-S. Among the ICP-M subgroups, the ICP-M_p_ exhibited the highest risk of adverse outcomes. Ursodeoxycholic acid (UDCA) treatment was found to be beneficial in reducing the risk of preterm birth, particularly in the ICP-M. The study also highlighted that late preterm or post-term delivery in the ICP-M patients exacerbates NICU risk.

**Conclusion:**

Women with ICP-M experience elevated perinatal risks, including a higher risk of coexisting GDM, as well as increased risks of preterm birth and NICU admissions. Personalized clinical management, optimizing delivery timing based on clinical subtypes, and providing UDCA to improve neonatal outcomes during pregnancy are important measures worthy of attention.

## Introduction

ICP is a pregnancy-specific complication that typically occurs in the second and third trimesters. Clinically, it presents with pruritus and elevated fasting serum total bile acids (sTBAs) (≥10 μmol/L), which may be accompanied by elevated liver enzymes, and resolves rapidly or returns to normal after delivery. ICP increases the morbidity and mortality of perinatal diseases, including preterm birth, fetal distress, and stillbirth (1). The incidence of ICP varies by ethnicity and region, with rates of 0.1–1.5% in Europe, the USA, Canada, and Australia (2), and as high as 4–10% in some areas of China, such as Chongqing, Sichuan, and the Yangtze River basin (3, 4).

The diagnostic criteria for ICP remain controversial due to the lack of specific clinical symptoms or biomarkers that exclusively indicate ICP because subjecting pregnant women to excessive medical examinations is unethical. The most widely accepted international diagnostic criteria for ICP follow the guidelines established by the Royal College of Obstetricians and Gynecologists (RCOG), which include elevated serum total bile acid levels (sTBA ≥10 μmol/L), abnormal liver function (elevated transaminase levels), and pruritus (skin itching, typically subsiding rapidly after delivery) as diagnostic markers (5). Contrary to typical expectations, a case of intrauterine fetal demise due to asymptomatic hypercholanemia was reported in Chengdu, China, indicating that elevated sTBA levels during pregnancy may pose a lethal risk to the fetus even in the absence of other typical symptoms (6). As a result, some hospitals have revised the standard diagnostic criteria, suggesting a more conservative categorization of pregnant women with elevated sTBA as having ICP, regardless of the presence of other symptoms (7). According to these criteria, all pregnant women with ICP undergo strict medical monitoring to maximally control the risk of fatal fetal harm. However, under this conservative diagnostic standard, the incidence of medically indicated preterm birth has significantly increased to avoid potential fetal demise, leading to an increase in adverse neonatal pregnancy outcomes and even affecting lifelong fetal development. Given these adverse reactions, there is an urgent need to determine whether this conservative diagnostic strategy is overly cautious or unnecessary. However, combining TBA levels with clinically recognizable typical symptoms to distinguish between the different risk levels of ICP patients is expected to bring significant clinical benefits. This approach may not only improve the identification rate of high-risk ICP patients but also effectively prevent the overtreatment of low-risk patients. Furthermore, considering the recognizability of the symptoms, this clinical stratification strategy is highly practical, enabling doctors to more accurately define high-risk ICP pregnancies and formulate personalized treatment plans based on the severity of the disease.

UDCA is widely recommended in the international guidelines for managing ICP (8), primarily targeting maternal symptom relief and biochemical improvement (9, 10). Recent systematic reviews consolidate evidence that UDCA effectively reduces pruritus and normalizes liver enzymes, although its impact on perinatal outcomes remains controversial (11). This therapeutic ambiguity underscores the need to evaluate the efficacy of UDCA across clinically distinct ICP subgroups, particularly multisymptomatic phenotypes (11). In addition, optimizing the timing of delivery is a key strategy to reduce the incidence of perinatal neonatal morbidity in singleton pregnancy ICP patients (12). In particular, given the variations in hospital environments and clinical practices across countries/regions, finding a balance between the risks of early delivery and the related risks of continuing pregnancy is challenging. To date, neither the Maternal Fetal Medicine Society nor the Obstetricians and Gynecologists Society has provided recommendations on the timing of delivery for ICP and its different subtypes. Pregnant women and their partners, clinicians, and guideline developers require reliable data to estimate the risks associated with continuing pregnancy and the neonatal risks of prematurity to determine the optimal timing of delivery.

This study aimed to explore the effectiveness of symptom-based clinical subtyping of ICP for identifying women at high risk for adverse perinatal outcomes and to further investigate the risk of perinatal outcomes associated with different symptom combinations in the ICP-M. In addition, the therapeutic effects of UDCA and second-line medications such as SAMe on ICP and its different clinical subtypes will be examined. Finally, the study will investigate the risk of severe adverse neonatal outcomes for different ICP subtypes at late preterm and term weeks of gestation, attempting to optimize the timing of delivery.

## Materials and methods

### Ethical approval

This study was approved by the ethics committee of Chongqing Medical University (ID: 20220627). To safeguard patient privacy, all personally identifiable information was removed from the cases, and all acquired data were kept anonymous.

### Study design

This retrospective cohort study was conducted from October 2018 to October 2021 at two tertiary and first-class hospitals in Chongqing, China, namely, the First Affiliated Hospital of Chongqing Medical University and the Women and Children’s Hospital of Chongqing Medical University. These two hospitals are the largest maternity hospitals in Chongqing, with the total number of neonates exceeding 10,000 and 15,000, respectively. We established an ICP specialized cohort involving two centers, with the study subjects being pregnant women diagnosed with ICP based on the criteria of sTBA levels ≥10 μmol/L, and/or elevated alanine aminotransferase (ALT) levels, and/or direct bilirubin levels exceeding the normal laboratory reference values, all measured in the fasting state. In these two centers, TBA is measured during routine prenatal visits or when symptoms such as unexplained pruritus arise. Before enrollment in the study, we extracted data solely from the electronic health records of pregnant women diagnosed with ICP, including their initial laboratory results at the time of ICP diagnosis, such as complete blood count, urinalysis, liver and kidney function tests, thyroid function tests, and TBA levels.

It is important to note that this retrospective study focuses solely on late preterm births (i.e., those occurring at or after 34 weeks of gestation). Because in our study, cases of preterm delivery before 34 weeks of gestation are relatively rare. Given the rarity of early preterm births, their inclusion could disproportionately influence the results and widen the confidence intervals in the analysis of restrictive cubic splines (RCS) for estimating optimal gestational week for delivery. By focusing on late preterm births, we aimed to provide a more reliable and precise estimation of optimal delivery timing, which is highly relevant for the majority of preterm cases in our study population. Therefore, including these cases may lead to inaccurate estimates of the confidence interval for the RCS, thereby affecting our conclusions regarding the more common group of pregnant women experiencing late preterm delivery and subsequent pregnancies. In addition, extreme cases of ICP may significantly interfere with comparative studies between the ICP-M and the ICP-S.

The inclusion criteria were as follows: (i) complete medical records; (ii) singleton pregnancy; and (iii) confirmed diagnosis of ICP. The exclusion criteria were as follows: (i) fetuses with chromosomal abnormalities with or without structural defects; (ii) women diagnosed with the COVID-19 virus; and (iii) delivery before 34 weeks of gestation. A total of 3,188 pregnant women met the inclusion criteria. After applying the exclusion criteria, 2,057 cases were ultimately included in the study. Maternal outcomes of interest included intrapartum blood loss, postpartum blood loss, GDM, gestational hypertension, preeclampsia, cervical laceration, vaginal laceration, and laceration perineum. Neonatal indicators included gestation week at delivery, birth weight, head circumference, abdominal girth, and fetal heart rate, while neonatal outcomes included premature delivery, low-birth-weight infant, hyperamniotic fluid, macrosomia, fetal growth restriction (FGR), fetal distress, amniotic fluid stool staining, and NICU admission.

### Data collection

All demographic and clinical data, including maternal information and outcomes as well as neonatal outcomes, were derived from the electronic medical records of Chongqing Medical University and the Women and Children’s Hospital of Chongqing Medical University. Two data collectors extracted the medical records simultaneously and reviewed and reconciled any discrepancies in their descriptions to ensure accurate data extraction. Furthermore, all data collectors were blinded to the primary objectives and hypotheses of this study.

The baseline biochemical indicators related to ICP mentioned here are all data from the first measurements taken at the time of diagnosis or after the diagnosis of ICP. The specific indicators include: albumin, globulin, total bilirubin, direct bilirubin, and indirect bilirubin. For ALT (alanine aminotransferase), aspartate aminotransferase (AST), and TBA, not only the values at diagnosis or the first recorded data after diagnosis but also the levels before delivery, as well as the corresponding pruritus status, were documented. In addition, the OGTT results presented at baseline include data from the following three time points: OGTT0, which is the fasting result; OGTT1, which is the result after 1 h; and OGTT2, which is the result after 2 h. These data were obtained during routine OGTT screening conducted at 24 to 28 weeks of pregnancy.

### Definition of ICP clinical subtypes and research outcomes of interest

In our study, ICP was the primary exposure. Given the controversy in diagnostic criteria, ICP was categorized into two clinical phenotypes based on different diagnostic standards. The first phenotype, diagnosed under a “narrow definition,” was termed “multisymptomatic ICP” (ICP-M), identified by elevated sTBA levels in conjunction with pruritus, elevated transaminases, or both. The second phenotype, diagnosed under a “broad definition,” was termed “single-symptom ICP” (ICP-S), recognized by elevated sTBA levels without liver dysfunction, pruritus, or other diagnosed hepatobiliary disorders. More specifically, ICP-M included various symptom combinations: (i) elevated TBA with elevated transaminase levels (ICP-M_T_), (ii) elevated sTBA with pruritus (ICP-M_P_), and (iii) elevated TBA with both elevated transaminase levels and pruritus (ICP-M_B_). Gestational age was defined using standard clinical practice for estimating gestational age: the best obstetric estimate recorded in the birth records, which is calculated based on the last menstrual period and other clinical and ultrasonographic parameters.

### Maternal outcomes

According to the International Association of Diabetes and Pregnancy Study Groups consensus panel (IADPSG/WHO), the definition of GDM is as follows: GDM is diagnosed in pregnant women at 24–28 weeks of gestation who, after fasting for 8–10 h, undergo a 75 g oral glucose tolerance test (OGTT) and meet any of the following criteria: fasting plasma glucose ≥5.1 mmol/L, 1-h plasma glucose ≥10.0 mmol/L, or 2-h plasma glucose ≥8.5 mmol/L (13). Hypertensive disorders of pregnancy (HDP) are defined as: according to the International Society for the Study of Hypertension in Pregnancy, a systolic blood pressure of ≥140 mm Hg and/or a diastolic blood pressure of ≥90 mm Hg on at least two occasions after 20 weeks of gestation, in the absence of significant proteinuria. Preeclampsia is diagnosed with hypertension and proteinuria of ≥300 mg in 24 h or a dipstick reading of “++” on at least two occasions in midstream or catheter urine samples within 24 h (14). The study also selected cervical lacerations, vaginal lacerations, and perineal lacerations as maternal outcome indicators because these lacerations are primarily associated with vaginal deliveries, which are generally avoided in cesarean sections. Therefore, the incidence and types of these lacerations can indirectly reflect the proportion of cesarean sections versus vaginal deliveries, serving as an important reference for assessing the mode of delivery.

### Neonatal outcomes

Preterm birth is defined as the delivery of an infant at less than 37 complete weeks of gestational age, calculated from the first day of the last menstrual period. Low birth weight (LBW) refers to a newborn weighing less than 2,500 g (5 pounds, 8 ounces) at birth, regardless of gestational age. Polyhydramnios is defined as an amniotic fluid volume (AFV) exceeding 2,000 mL (15, 16). Macrosomia is characterized by a birth weight greater than 4,000 g. FGR refers to a fetal weight below the third percentile. Amniotic fluid stool staining refers to the presence of meconium, which is the fetal stool, in the amniotic fluid during delivery. NICU admission denotes the process whereby a newborn is admitted to the neonatal intensive care unit for specialized treatment and care due to health issues present at or within the first 28 days of life. All outcome measures are aligned with and derived from previous large-scale studies on comparative programs and national guidelines, reflecting the applicability and extensibility of the research (17, 18).

### Statistical analysis

The statistical analysis was conducted using SPSS software version 26.0 and R Studio version 4.0.2. Subjects were initially stratified based on the presence or absence of clinical symptoms. To describe the sociodemographic characteristics of the subjects, continuous data conforming to a normal distribution were presented as mean values and standard deviations, while non-normally distributed data were represented by median and interquartile range (IQR). Univariate analysis was first performed on the continuous and categorical demographic data as well as related liver and glycemic biochemical markers for the ICP-S and ICP-M groups. Categorical variables were expressed as counts and frequency distributions. Continuous variables were compared using the Mann–Whitney U-test, whereas categorical variables were analyzed using the chi-square or Fisher’s exact test. Subsequently, univariate and multivariate logistic regression analyses were employed to estimate the odds ratios of perinatal outcomes in the ICP-M group compared to the ICP-S group. Although the temporal relationship between ICP and GDM cannot be definitively determined, in this study, GDM was treated as an independent variable to facilitate comparison between different ICP subtypes. Key confounding factors, including maternal age, pre-gestational BMI, nulliparity, timing of diagnosis, and IVF history, were accounted for in the analysis. Further subgroup analysis was conducted for the ICP-M to examine the risks of adverse maternal and neonatal outcomes associated with different combinations of clinical symptoms in the ICP-M subtypes. The analysis was performed using the Kruskal–Wallis test for continuous variables and the chi-square test for categorical variables. After adjusting for confounding factors, multivariate logistic regression analyses were conducted to estimate the ORs and 95% CIs for NICU admission across different gestational weeks at delivery, using 37 weeks of gestation as the reference point for the intersection of late preterm and full-term births. The analyses were performed across all pregnant women, the ICP-M group, and the ICP-S group. Finally, the RCS were utilized to examine whether there was a non-linear relationship between gestation week at delivery and the incidence of severe neonatal adverse outcomes, specifically NICU admission, within the entire cohort of ICP, the ICP-M group, and the ICP-S group, and to observe the trend of ORs.

## Results

### General clinical characteristics of the ICP-S group and the ICP-M group

**Figure 1:**
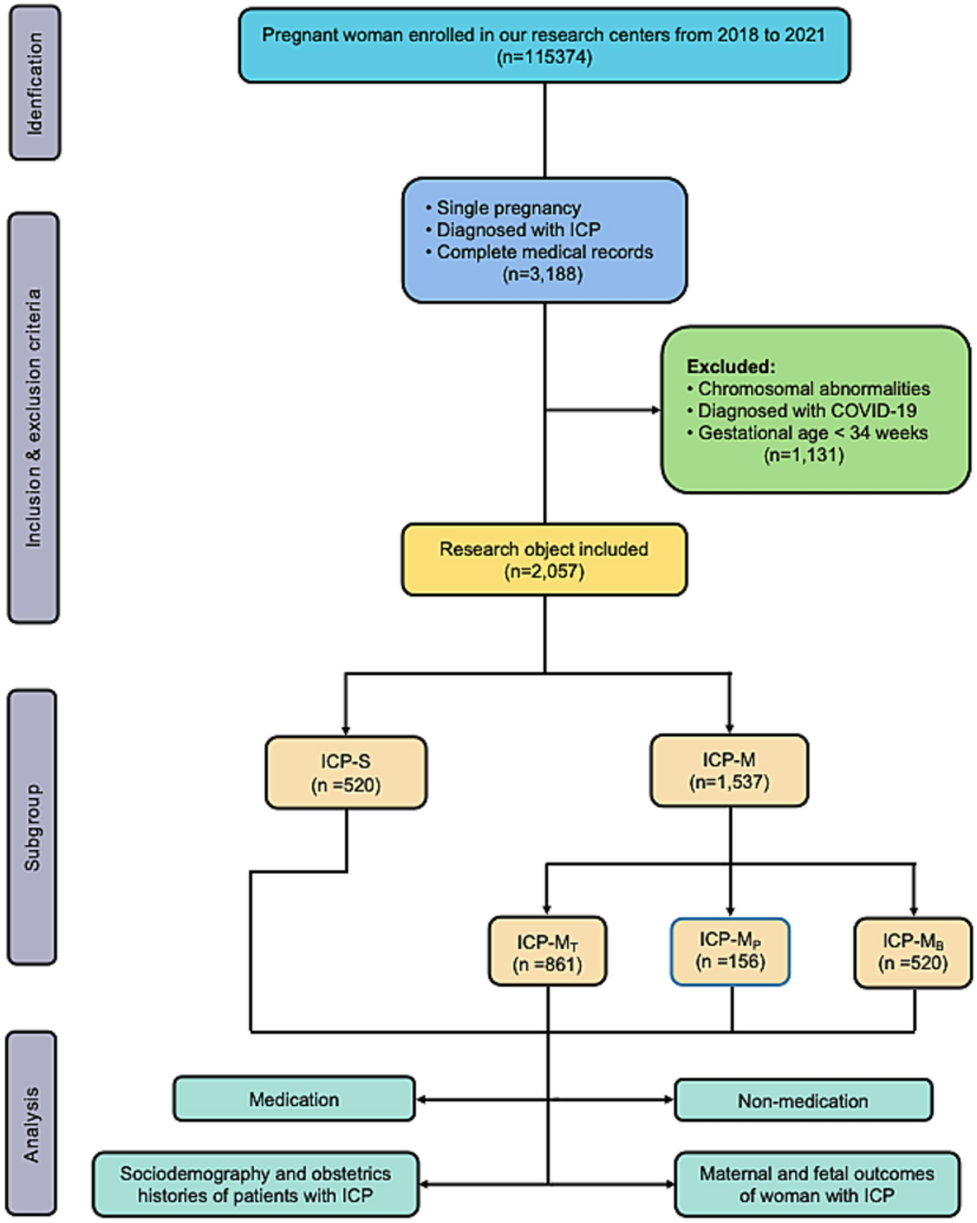
Flowchart of this retrospective cohort study.

Between 2017 and 2021, we assessed the eligibility of 115,374 singleton pregnancy cases at the First Affiliated Hospital of Chongqing Medical University and the Chongqing Medical University Affiliated Women and Children’s Hospital. After applying exclusion criteria, a total of 2,057 singleton pregnancy cases with ICP were included in the final analysis (Figure 1). Of these cases, 520 (25.28%) were categorized into the ICP-S group, while 1,537 (74.72%) were allocated to the ICP-M group.

The analysis of baseline characteristics between the two groups revealed that among women with ICP, the rates of IVF (*p* < 0.001) and nullipara (*p* = 0.029) are higher in the ICP-M group (Table 1). In particular, the diagnosis of ICP was made earlier in the ICP-M group than the ICP-S group (36.00, IQR 32.86–38.00 vs. 37.00, IQR 33.86–38.00). In terms of biochemical markers, the liver function-related indicators in the ICP-M pregnant women, including levels of ALT, AST, TBA, albumin, globulin, total bilirubin, and direct bilirubin, were significantly higher than those in the ICP-S pregnant women. This finding is consistent with the criteria used to classify these groups, as the elevated liver function indicators were part of the basis for distinguishing ICP-M from ICP-S. In addition, the ICP-M group also demonstrated significantly higher blood glucose values at all time points during the OGTT (FBG, 4.44, IQR 4.20–4.70 vs. 4.40, IQR 4.20–4.60, *p* = 0.006; OGTT1h 7.90, IQR 6.70–9.30 vs. 7.70, IQR 6.41–8.90, *p* = 0.002; OGTT2h 6.70, IQR 5.70–7.90 vs. 6.50, IQR 5.63–7.50, *p* = 0.003). However, these differences, while statistically significant, are relatively minor and may not be clinically meaningful. Consequently, women with ICP-M were more likely to require second-line medications post-diagnosis, such as S-adenosylmethionine (SAMe) (7.48% vs. 2.50%, *p* < 0.001). Although there was no significant difference in the dosage of UDCA, the usage rate was still higher in the ICP-M group (38.32% vs. 33.65%, *p* = 0.057). Furthermore, there were no statistically significant differences between the two groups in terms of age, preconception BMI, smoking, drinking, nulligravida status, hepatitis history, placental implantation, and other biochemical markers (Table 1).

**Table 1:** Baseline characteristics of patients diagnosed with different clinical subtypes of ICP from 2018 to 2021.

Characteristics	ICP (*n* = 2,057)	ICP-S (*n* = 520)	ICP-M (*n* = 1,537)	*p*-value
Maternal age (years), median (IQR)	30.00 (27.00, 32.00)	30.00 (27.00, 32.00)	30.00 (27.00, 32.00)	0.544
Pre-gestation BMI (kg/m^2^), median (IQR)	20.70 (19.14, 22.66)	20.62 (19.03, 22.58)	20.70 (19.15, 22.76)	0.222
Weeks of diagnosis (weeks), median (IQR)	36.4 (33.0, 38.3)	37.0 (33.9, 38.9)	36.0 (32.9, 38.0)	<0.001*
IVF, *n* (%)	281 (13.66)	56 (10.77)	225 (14.64)	0.026*
Smoking, *n* (%)	23 (1.12)	7 (1.35)	16 (1.04)	0.567
Drinking, *n* (%)	14 (0.68)	5 (0.96)	9 (0.59)	0.510
Nulligravida, *n* (%)	1, 128 (54.84)	303 (58.27)	825 (53.68)	0.069
Nullipara, *n* (%)	1, 454 (70.69)	348 (66.92)	1, 106 (71.96)	0.029*
Hepatitis history, *n* (%)	227 (11.04)	59 (11.35)	168 (10.93)	0.794
UDCA, *n* (%)	764 (37.14)	175 (33.65)	589 (38.32)	0.057
SAMe, *n* (%)	128 (6.22)	13 (2.50)	115 (7.48)	<0.001*
ALT (U/L), median (IQR)	66.00 (20.00, 138.00)	13.00 (10.00, 20.00)	98.00 (56.00, 174.00)	<0.001*
AST (U/L), median (IQR)	49.00 (25.00, 95.00)	20.00 (16.00, 25.00)	66.00 (43.00, 116.00)	<0.001*
OGTT0 (mmol/L), median (IQR)	4.40 (4.20, 4.70)	4.40 (4.20, 4.60)	4.44 (4.20, 4.70)	0.006*
OGTT1 (mmol/L), median (IQR)	7.80 (6.60, 9.20)	7.70 (6.41, 8.90)	7.90 (6.70, 9.30)	0.002*
OGTT2 (mmol/L), median (IQR)	6.60 (5.70, 7.80)	6.50 (5.63, 7.50)	6.70 (5.70, 7.90)	0.003*
Total protein (g/L), median (IQR)	65.00 (62.00, 69.00)	65.00 (62.00, 68.00)	66.00 (62.00, 69.00)	0.501
Albumin (g/L), median (IQR)	34.00 (32.00, 36.00)	34.00 (32.00, 36.00)	34.00 (32.00, 36.00)	0.007*
Globulin (g/L), median (IQR)	31.00 (29.00, 34.00)	31.00 (28.00, 33.00)	31.00 (29.00, 34.00)	0.005*
Total bilirubin (μmol/L), median (IQR)	8.20 (4.50, 11.40)	7.20 (2.90, 9.70)	8.60 (5.10, 12.10)	<0.001*
Direct bilirubin (μmol/L), median (IQR)	3.80 (1.90, 7.10)	2.40 (1.50, 6.10)	4.20 (2.10, 7.50)	<0.001*
Indirect bilirubin (μmol/L), median (IQR)	8.00 (5.70, 12.50)	8.00 (5.80, 12.50)	7.90 (5.70, 12.50)	0.923
TBA (μmol/L), median (IQR)	18.00 (12.80, 29.00)	15.90 (12.20, 23.20)	18.90 (13.30, 31.70)	<0.001*
TBA is between 40 and 100, *n* (%)	272 (13.22)	41 (7.89)	231 (15.03)	
TBA is greater than 100, *n* (%)	33 (1.60)	0 (0.00)	33 (2.15)	

ICP, Intrahepatic cholestasis of pregnancy; ICP-S, single-symptom ICP; ICP-M, multisymptomatic ICP; IVF, in vitro fertilization; UDCA, ursodeoxycholic acid; SAMe, S-adenosylmethionine; ALT, alanine aminotransferase; AST, aspartate aminotransferase; OGTT, oral glucose tolerance test; TBA, total bile acids.

Direct bilirubin (conjugated bilirubin) is the water-soluble bilirubin formed after conjugation in the liver, primarily used to assess liver conjugation and excretion functions; indirect bilirubin (unconjugated bilirubin) is the lipophilic bilirubin that has not undergone hepatic conjugation, mainly used to evaluate the degree of red blood cell destruction and liver metabolic function.

*p*-values were calculated using the Mann–Whitney *U*-test for continuous variables presented as median (IQR) and the chi-square test for categorical variables presented as *n* (%). Differences marked with * indicate significance at *p* < 0.05.

### Comparison of maternal and neonatal outcomes between the ICP-S and the ICP-M groups

To investigate whether ICP-M leads to more adverse perinatal outcomes than ICP-S, a comparative analysis of perinatal outcomes for both groups of pregnant women and their newborns was conducted (Table 2). In terms of maternal outcomes, univariate analysis revealed that the ICP-M group had a significantly higher rate of concurrent GDM than the ICP-S group. This increased rate persisted even after adjusting for potential confounders such as maternal age, pre-gestation BMI, nullipara, weeks of diagnosis, and IVF, with the ICP-M group having a significantly elevated occurrence of concurrent GDM (aOR 1.57, 95% CI 1.23–2.01). However, there were no significant differences between the two groups in clinical maternal outcomes such as intrapartum blood loss, postpartum blood loss, gestational hypertension, preeclampsia, cervical laceration, vaginal laceration, and laceration of the perineum. In addition, before delivery, there were significant differences in the characteristics and disease severity between the ICP-M group and the ICP-S group. Specifically, the TBA levels before delivery were significantly higher in the ICP-M group than in the ICP-S group, with median values of 14.80 (10.10, 26.30) μmol/L and 12.90 (9.50, 21.30) μmol/L, respectively (*p* < 0.001). Moreover, the proportion of patients in the ICP-M group who continued to be classified as ICP-M before delivery was significantly higher than that in the ICP-S group, at 65.32 and 4.62%, respectively (*p* < 0.001) (Supplementary Table 2).

**Table 2:** Maternal and neonatal outcomes in patients with different clinical subtypes of ICP.

Outcomes	ICP-Total (*n* = 2,057)	ICP-S (*n* = 520)	ICP-M (*n* = 1,537)	*p*-value
Maternal outcome				
Intrapartum blood loss (mL), median (IQR)	300.00 (220.00, 400.00)	300.000 (210.00, 400.00)	300.00 (220.00, 400.00)	0.691
Postpartum blood loss (mL), median (IQR)	45.00 (20.00, 80.00)	45.000 (23.00, 85.00)	41.000 (20.00, 80.00)	0.139
Gestational diabetes, *n* (%)	530 (25.77)	101 (19.42)	429 (27.91)	<0.001*
Gestational hypertension, *n* (%)	67 (3.26)	11 (2.12)	56 (3.64)	0.090
Preeclampsia, *n* (%)	117 (5.69)	31 (5.96)	86 (5.60)	0.755
Cervical laceration, *n* (%)	55 (2.67)	12 (2.31)	43 (2.80)	0.549
Vaginal laceration, *n* (%)	207 (10.06)	53 (10.19)	154 (10.02)	0.910
Laceration perineum, *n* (%)	215 (10.45)	61 (11.73)	154 (10.02)	0.270
Neonatal outcome				
Weeks of diagnosis (weeks), median (IQR)	38.0 (37.0, 39.0)	38.4 (37.1, 39.3)	38.0 (36.9, 39.0)	<0.001*
Premature delivery, *n* (%)	338 (16.43)	53 (10.19)	285 (18.54)	<0.001*
Birth weight (g), median (IQR)	3090.00 (2730.00, 3420.00)	3140.00 (2800.00, 3470.00)	3070.00 (2700.00, 3400.00)	<0.001*
Low-birth-weight infant, *n* (%)	281 (13.66)	47 (9.04)	234 (15.22)	<0.001*
Head circumference (cm), median (IQR)	33.00 (32.16, 34.00)	33.32 (32.40, 34.00)	33.00 (32.06, 34.00)	<0.001*
Abdominal girth (cm), median (IQR)	330.00 (32.00, 34, 08)	33.00 (32.00, 34.28)	33.00 (31.80, 34.00)	0.003*
Fetal heart rate (bpm), median (IQR)	140.00 (139.00, 146.00)	140.00 (138.00, 146.00)	140.00 (139.00, 145.00)	0.333
Hyperamniotic fluid, *n* (%)	51 (2.48)	7 (1.35)	44 (2.86)	0.055
Macrosomia, *n* (%)	54 (2.63)	18 (3.46)	36 (2.34)	0.168
FGR, *n* (%)	37 (1.80)	7 (1.35)	30 (1.95)	0.369
Fetal distress, *n* (%)	166 (8.07)	45 (8.65)	121 (7.87)	0.572
NICU admission, *n* (%)	187 (9.09)	34 (6.54)	153 (9.95)	0.019*
Amniotic fluid stool staining, *n* (%)	331 (16.09)	84 (16.15)	247 (16.07)	0.964

ICP, intrahepatic cholestasis of pregnancy; ICP-S, single-symptom ICP; ICP-M, multisymptomatic ICP; FGR, fetal growth restriction; NICU admission, neonatal intensive care unit admission.

*p*-values were calculated using the Mann–Whitney *U*-test for continuous variables presented as median (IQR) and the chi-square test for categorical variables presented as *n* (%). Differences marked with * indicate significance at *p* < 0.05.

Regarding neonatal outcomes, the ICP-M group had a significantly earlier gestational week at delivery and an increased risk of preterm birth (a gestational week at delivery 38.0, 36.9–39.0 vs. 38.4, 37.1–39.3; premature delivery 18.54% vs. 10.19%). In addition, newborn measurements in the ICP-M group were significantly lower than those in the ICP-S group, including birth weight (3070.00, 2700.00–3400.00 vs. 3140.00, 2800.00–3470.00), head circumference (33.00, 32.06–34.00 vs. 33.32, 32.40–34.00), and abdominal girth (33.00, 31.80–34.00 vs. 33.00, 32.00–34.28). After adjusting for confounders, the risk of preterm birth in the ICP-M group remained significantly increased (aOR 1.92, 95% CI 1.41–2.67) as well as the risks of low birth weight (aOR 1.81, 95% CI 1.30–2.52) and NICU admission (aOR 1.48, 95% CI 1.01–2.22). However, there were no statistically significant differences between the two groups in neonatal outcomes such as fetal heart rate abnormalities, polyhydramnios, macrosomia, FGR, fetal distress, and meconium-stained amniotic fluid (Supplementary Table 1).

### Comparison of maternal and neonatal outcomes among different clinical symptom subgroups of ICP-M

To gain a deeper understanding of the clinical heterogeneity of ICP-M, a subgroup analysis was first conducted. Based on different combinations of symptoms, the study subjects were divided into three subgroups: the ICP-M_T_ group with elevated serum TBA and high aminotransferase levels, the ICP-M_P_ group with high TBA and pruritus, and the ICP-M_B_ group with high TBA, elevated aminotransferase levels, and pruritus. In terms of maternal outcomes, the ICP-M_B_ group had a significantly higher risk of coexisting GDM than the ICP-M_P_ group (30.12% vs. 16.88%, *p* = 0.005). However, the risks of vaginal and perineal lacerations during childbirth in the ICP-M_B_ group were unexpectedly lower than those in the ICP-M_P_ group (vaginal laceration 6.76% vs. 14.94%, *p* = 0.003; perineal laceration 7.14% vs. 13.64%, *p* = 0.019). This finding may be partly attributed to the lower proportion of vaginal births in the ICP-M_B_ group. In addition, significant differences were observed in intrapartum and postpartum bleeding among the different symptom combinations of ICP-M. Before delivery, significant differences in characteristics and disease severity were also observed among different subgroups of ICP-M patients. Specifically, the TBA levels were significantly higher in the ICP-M_B_ and ICP-M_T_ groups than the ICP-M_P_ group, with median values of 15.30 (10.10, 28.20) μmol/L and 15.10 (10.40, 25.40) μmol/L, respectively, compared with 11.70 (7.30, 23.80) μmol/L in the ICP-MP group (*p* < 0.001). In addition, the proportion of patients continuing to be classified as ICP-M before delivery was significantly higher in the ICP-MB group than in the ICP-M_T_ and ICP-M_P_ groups, at 76.15, 59.81, and 59.62%, respectively (*p* < 0.001). These findings suggest that patients in the ICP-M_B_ group had more severe disease before delivery (Supplementary Table 3).

Regarding neonatal outcomes, the ICP-M_B_ group had significantly higher risks than the ICP-M_T_ group in terms of premature delivery (26.45% vs. 14.12%, *p* < 0.001), low-birth = weight infants (21.04% vs. 11.90%, p < 0.001), polyhydramnios (4.44% vs. 1.98%, *p* = 0.030), and NICU admissions (12.74% vs. 8.64%, *p* = 0.039). These results suggest that newborns in the ICP-M_B_ group may face more perinatal health challenges. Furthermore, the rate of premature delivery in the ICP-M_B_ group was also significantly higher than in the ICP-M_P_ group (Table 3).

**Table 3:** Maternal and neonatal outcomes of patients with various symptom combination subtypes of ICP-M.

Outcomes	ICP-M_T_ (*n* = 861)	ICP-M_P_ (*n* = 156)	ICP-M_B_ (*n* = 520)	*p*-value
Maternal outcome				
Intrapartum blood loss^b^ (mL), Median (IQR)	300.00 (200.00, 400.00)	300.00 (220.00, 400.00)	300.00 (300.00, 400.00)	0.020*
Postpartum blood loss (mL), Median (IQR)	45.00 (20.00, 80.00)	50.00 (30.00, 85.00)	40.000 (20.00, 80.00)	0.006*
Gestational diabetes^a^, *n* (%)	241 (28.12)	26 (16.88)	156 (30.12)	0.005*
Gestational hypertension, *n* (%)	35 (4.08)	6 (3.90)	15 (2.90)	0.517
Preeclampsia, *n* (%)	54 (6.30)	7 (4.55)	25 (4.83)	0.428
Cervical laceration, *n* (%)	29 (3.38)	6 (3.90)	8 (1.54)	0.094
Vaginal laceration^a^^,^^b^, *n* (%)	96 (11.20)	23 (14.94)	35 (6.76)	0.003*
Laceration perineum^a^, *n* (%)	94 (10.97)	21 (13.64)	37 (7.14)	0.019*
Neonatal outcome				
Weeks of gestation^a^^,^^b^, median (IQR)	38.3 (37.0, 39.1)	38.0 (37.0, 39.0)	37.7 (36.0, 38.7)	<0.001*
Premature delivery^a^^,^^b^, *n* (%)	121 (14.12)	26 (16.88)	137 (26.45)	<0.001*
Birth weight^b^ (g), median (IQR)	3120.00 (2780.00, 3460.00)	3050.00 (2715.00, 3390.00)	2980.00 (2570.00, 3320.00)	<0.001*
Low-birth-weight infant^b^, *n* (%)	102 (11.90)	22 (14.29)	109 (21.04)	<0.001*
Head circumference^b^ (cm), median (IQR)	33.16 (32.30, 34.00)	33.00 (32.18, 33.89)	32.75 (31.79, 33.70)	<0.001*
Abdominal girth (cm), median (IQR)	33.00 (32.00, 34.20)	33.00 (31.84, 34.00)	32.80 (31.40, 34.00)	0.002*
Fetal heart rate (bpm), median (IQR)	140.00 (138.00, 146.00)	140.000 (139.00, 145.00)	140.00 (139.00, 145.00)	0.853
Hyperamniotic fluid^b^, *n* (%)	17 (1.98)	4 (2.60)	23 (4.44)	0.030*
Macrosomia, *n* (%)	26 (3.03)	3 (1.95)	7 (1.35)	0.129
FGR, *n* (%)	19 (2.22)	4 (2.60)	7 (1.35)	0.445
Fetal distress, *n* (%)	75 (8.75)	9 (5.84)	37 (7.14)	0.341
NICU admission^b^, *n* (%)	74 (8.64)	13 (8.44)	66 (12.74)	0.039*
Amniotic fluid stool staining, *n* (%)	139 (16.22)	24 (15.58)	84 (16.22)	0.980

a Significant difference between ICP-M_P_ and ICP-M_B_.

b Significant difference between ICP-M_T_ and ICP-M_B_.

ICP, Intrahepatic cholestasis of pregnancy; ICP-M_T_, high sTBA with only high transaminase levels ICP; ICP-M_P_, high sTBA with only pruritus ICP; ICP-M_B_, high sTBA with both high transaminase levels and pruritus ICP; FGR, fetal growth restriction; NICU admission, neonatal intensive care unit admission.

*p*-values were calculated using the Kruskal–Wallis test for continuous variables presented as median (IQR) and the chi-square test for categorical variables presented as *n* (%). Differences marked with * indicate significance at *p* < 0.05.

### Pharmacological impacts on pregnant women with different subtypes of ICP

To investigate the impact of pharmacotherapy on pregnancy outcomes in pregnant women with different clinical subtypes of ICP, significant maternal and neonatal indicators and outcomes from various subtypes were selected for further analysis. Initially, the effect of first-line medication UDCA or second-line medications such as SAMe on GDM was analyzed. The results indicated that neither UDCA nor SAMe showed a significant improvement compared to ICP pregnant women who did not receive medication. In the analysis of neonatal indicators and outcomes, both in the overall ICP pregnant cohort and within different clinical subtypes of ICP, the weeks of gestation at delivery and neonatal birth parameters (such as weight, head circumference, and abdominal circumference) were significantly lower in women treated with UDCA and second-line medications than those who did not receive treatment. This suggests that pregnant women with ICP who received medication may have a higher severity of disease and that pharmacotherapy may not fully mitigate the risk of these adverse outcomes. Similarly, the risk of preterm birth was significantly higher in pregnant women with overall ICP and ICP-M who received second-line medications than non-users (ICP total 29.69% vs. 15.55%, *p* < 0.001; ICP-M 30.44% vs. 17.58%, *p* < 0.001). This finding may indicate that second-line medications are potentially less effective in preventing preterm birth in ICP pregnancies. However, it is also possible that the higher occurrence of preterm birth despite the addition of second-line agents reflects the severity of the ICP rather than the ineffectiveness of the second-line medications themselves. In particular, there was no significant difference in the risk of preterm birth between ICP subtypes treated with UDCA and those untreated, suggesting that UDCA as a first-line treatment may have a better effect in reducing or offsetting the risk of preterm birth in ICP-M pregnancies than SAMe. As for the rate of NICU admissions, no significant difference was found between ICP pregnant women treated with either UDCA or second-line medications compared to those who were untreated (Table 4). Moreover, the results showed that in the ICP-M group, patients who received UDCA treatment had significantly lower TBA levels before delivery than those who did not, with median values of 13.80 (8.60, 25.40) μmol/L and 15.80 (10.70, 26.80) μmol/L, respectively (*p* < 0.001). Moreover, the proportion of patients continuing to be classified as ICP-M before delivery was also significantly lower in the UDCA treatment group than in the untreated group (56.88% vs. 70.57%, *p* < 0.001). However, SAMe treatment did not show significant improvement in TBA levels or disease classification in the ICP-M group. In the ICP-S group, neither UDCA nor SAMe treatment significantly improved the characteristics or disease severity before delivery (Supplementary Table 4).

**Table 4:** Maternal and neonatal outcomes in patients with different clinical subtypes of ICP treated with ursodeoxycholic acid and second-line medication SAMe.

	ICP-Total						ICP-S						ICP-M					
Outcomes	UDCA		*p*-value	SAMe		*p*-value	UDCA		*p*-value	SAMe		*p*-value	UDCA		*p*-value	SAMe		*p*-value
	Take	Not take		Take	Not take		Take	Not take		Take	Not take		Take	Not take		Take	Not take	
Gestational diabetes, *n* (%)	206 (26.96)	324 (25.06)	0.340	36 (28.13)	494 (25.61)	0.529	34 (19.43)	67 (19.42)	0.998	2 (15.39)	99 (19.53)	0.709	172 (29.20)	257 (27.11)	0.374	34 (29.57)	395 (27.78)	0.681
Weeks of diagnosis (weeks), median (IQR)	38.0 (36.7, 38.9)	38.3 (37.0, 39.1)	<0.001*	37.3 (36.0, 38.6)	38.1 (37.0, 39.0)	<0.001*	38.0 (37.0, 38.9)	38.7 (37.4, 39.6)	<0.001*	37.1 (36.0, 38.7)	38.4 (37.3, 39.4)	0.008*	38.0 (36.4, 38.9)	38.1 (37.0, 39.1)	<0.001*	37.43 (36.0, 38.6)	38.0 (37.0, 39.0)	<0.001*
Premature delivery, *n* (%)	141 (18.46)	197 (15.24)	0.057	38 (29.69)	300 (15.55)	<0.001*	21 (12.00)	32 (9.275)	0.332	3 (23.08)	50 (9.86)	0.120	120 (20.37)	165 (17.41)	0.145	35 (30.44)	250 (17.58)	<0.001*
Birth weight (g), Median (IQR)	3020.00 (2680.00, 3340.00)	3130.00 (2760.00, 3460.00)	<0.001	2930.00 (2625.00, 3340.00)	3100.00 (2740.00, 3420.00)	0.004*	3000.00 (2720.00, 3360.00)	3250.00 (2860.00, 3550.00)	<0.001*	2760.00 (2540.00, 3030.00)	3160.00 (2820.00, 3470.00)	0.014*	3025.00 (2680.00, 3340.00)	3105.00 (2720.00, 3430.00)	0.011*	2930.00 (2640.00, 3350.00)	3080.00 (2700.00, 3410.00)	0.067
Low-birth-weight infant, *n* (%)	113 (14.79)	168 (12.99)	0.251	23 (17.97)	258 (13.38)	0.143	19 (10.86)	28 (8.12)	0.303	3 (23.08)	44 (8.68)	0.074	94 (15.96)	140 (14.77)	0.527	20 (17.39)	214 (15.05)	0.501
Head circumferencec (cm), median (IQR)	32.90 (32.00, 33.70)	33.20 (32.28, 34.00)	<0.001	32.60 (31.57, 33.50)	33.09 (32.20, 34.00)	<0.001*	33.08 (32.17, 34.00)	33.50 (32.50, 34.10)	<0.001*	32.48 (31.37, 34.20)	33.33 (32.43, 34.00)	0.149	32.84 (32.00, 33.60)	33.12 (32.17, 34.00)	<0.001*	32.60 (31.60, 33.50)	33.00 (32.10, 34.00)	<0.001*
Abdominal girth (cm), median (IQR)	32.97 (31.68, 34.00)	33.00 (32.00, 34.18)	0.002*	32.79 (31.40, 33.70)	33.00 (32.00, 34.10)	0.005*	33.00 (32.00, 33.95)	33.20 (32.04, 34.50)	0.005*	32.00 (30.50, 32.81)	33.00 (32.00, 34.37)	0.004*	32.92 (31.50, 34.00)	33.00 (32.00, 34.00)	0.046*	32.85 (31.40, 33.80)	33.00 (31.84, 34.00)	0.083
NICU admission, *n* (%)	69 (9.03)	118 (9.13)	0.942	12 (9.38)	175 (9.07)	0.908	12 (6.86)	22 (6.38)	0.834	0 (0.00)	34 (6.71)	NA	57 (9.68)	96 (10.13)	0.775	12 (10.44)	141 (9.92)	0.858

ICP, intrahepatic cholestasis of pregnancy; ICP-S, single-symptom ICP; ICP-M, multisymptomatic ICP; NICU admission, neonatal intensive care unit admission; UDCA, ursodeoxycholic acid; SAMe, S-adenosylmethionine.

*p*-values were calculated using the Mann–Whitney *U*-test for continuous variables presented as median (IQR) and the chi-square test for categorical variables presented as *n* (%). Differences marked with * indicate significance at *p* < 0.05.

### Identifying the optimal gestational week for delivery based on NICU admission rates among ICP subtypes

To elucidate the impact of gestational week at delivery on the incidence of severe neonatal adverse outcomes, specifically NICU admission, among different ICP subtypes, frequencies of various conditions and NICU admissions were categorized according to weeks of gestation for the ICP-S and ICP-M groups (Supplementary Table 5). It should be noted that the sample size for the ICP-S group is relatively small, which may limit the reliability of the findings in this subgroup. Within the gestational week range of 35^0^–35^6^ days, the NICU admission rate for the ICP-M group was significantly higher than that for the ICP-S group (*p* = 0.047). Using the late preterm and full-term boundary of 37 weeks as a reference, a multivariable logistic regression analysis further assessed the risk of NICU admission in relation to the gestational week at delivery for both the ICP-S and ICP-M groups, adjusting for confounding factors including maternal age, pre-pregnancy BMI, nullipara, and IVF in the model.

The study findings revealed that, with 37 weeks as the reference point due to its significance as the boundary between late preterm and term pregnancy, within the ICP-S group, compared to delivery at 37 weeks, the risk of NICU admission was significantly increased at 34, 35, and 36 weeks of gestation (aOR: 39.89, 95% CI: 6.08–791.20; aOR: 19.80, 95% CI: 2.69–403.22; aOR: 33.97, 95% CI: 6.24–634.22), while no significant difference in risk was observed at 38, 39, and 40 weeks, suggesting that ICP-S pregnancies may consider delivery upon reaching full term. However, for ICP-M pregnancies, in addition to a significant increase in NICU admission risk at 34, 35, and 36 weeks (aOR: 9.00, 95% CI: 4.75–17.64; aOR: 9.53, 95% CI: 5.25–18.05; aOR: 3.10, 95% CI: 1.67–5.94), a significant reduction in risk was noted at 38 and 39 weeks (aOR: 0.30, 95% CI: 0.11–0.70; aOR: 0.43, 95% CI: 0.17–0.99). Compared to delivery at 37 weeks, there was no significant difference in NICU admission risk when delivery occurred at 40 weeks (aOR: 0.97, 95% CI: 0.37–2.31) (Supplementary Table 6), indicating a potential low-risk delivery window for ICP-M pregnancies between 38 and 40 weeks.

**Figure 2:**
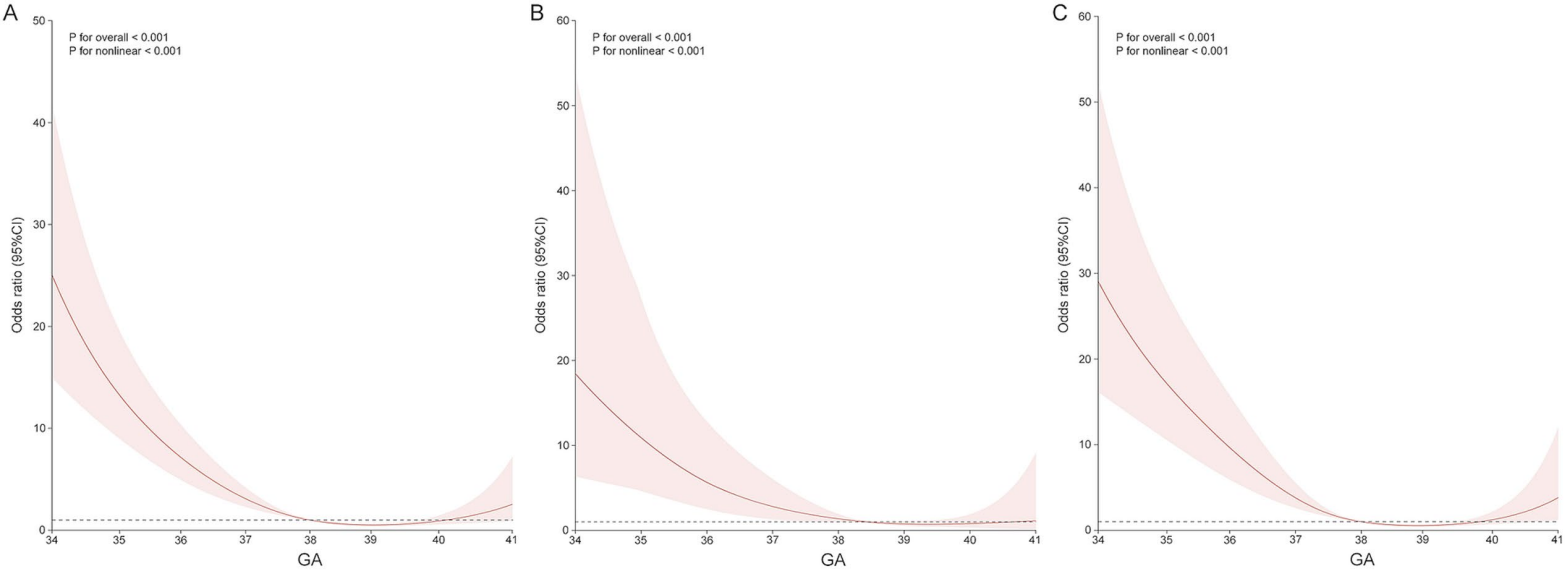
Association between delivery in singleton pregnancies with ICP and the odds ratio of NICU admission, fitted with restricted cubic splines. **(A)** All ICP pregnancies. **(B)** ICP-S pregnancies. **(C)** ICP-M pregnancies. The reference level for OR is the median gestational age at delivery. The reference line is Y = 1. These curves are adjusted for maternal age, pre-gestation BMI, nullipara, weeks of diagnosis, and IVF.

To further visualize the impact of gestational week at delivery on the incidence of severe neonatal adverse outcomes, specifically NICU admission, this study employed the analysis of RCS for both the ICP-S and ICP-M groups. The results indicated a non-linear relationship between gestational week and NICU admission in the ICP-M group (*p* for non-linearity <0.001). During the window period of 38–40 weeks of gestation, the risk of NICU admission is at its lowest; however, after 40 weeks of gestation, the risk of requiring neonatal intensive care is similar to that of late preterm infants. This indicates that the risk of NICU admission increases again after this window period of pregnancy. In contrast, within the ICP-S group, the risk of NICU admission remained relatively stable with increasing gestation week at delivery during full-term deliveries, without a clear cut-off point. This indicates that the ICP-S group does not have a significant window period like the ICP-M group. Given the limited number of deliveries after 40-week gestation in both the ICP-M and ICP-S groups (totaling only 9 cases), the wide 95% confidence interval underscores the potential uncertainty in estimating NICU admission risk for these late-term births. Thus, any conclusions drawn regarding NICU admission risk beyond 40 weeks should be approached with caution (Figure 2).

## Discussion

In the ongoing global debate regarding the diagnostic criteria for ICP, this study proposes a clinical classification method that aligns with international standards by combining TBA levels with typical clinical symptoms to distinguish between different risk levels of ICP patients. The primary advantage of this method lies in its ability to not only enhance the identification rate of high-risk ICP patients but also effectively avoid overtreatment of low-risk patients. However, we emphasize that this classification method should not solely rely on TBA values but should consider a more comprehensive look at clinical characteristics, such as liver enzyme levels or easily observable symptoms like pruritus. In this study, the TBA levels in the ICP-M group were significantly higher than those in the ICP-S group, which may relate to differences in TBA concentrations; however, current research has overly focused on establishing TBA thresholds for determining the severity of ICP. In clinical practice, physicians should prioritize these obvious clinical characteristics, rather than getting bogged down in the absolute relationship with contentious TBA thresholds, allowing for quicker and simpler identification of high-risk populations in clinical settings. Only in this way can we provide personalized treatment plans for patients and enhance the efficiency and effectiveness of clinical diagnosis and treatment.

Considering the higher incidence of preterm births in the ICP-M group, this group has a significantly greater relative risk of low birth weight than the ICP-S group, further confirming that the likelihood of adverse pregnancy outcomes in ICP-M mothers is indeed higher than in ICP-S patients. However, potential confounding factors, such as the use of antidiabetic medications, dietary habits, and physical activity levels, were not fully controlled for. Therefore, our findings should be interpreted with caution and may require validation in larger, prospective studies. The results also indicate an increased risk of comorbidity with GDM in the ICP-M group, leading to a significant rise in the risk of NICU admission, while associated neonatal metrics have also significantly declined. These findings underscore the clinical necessity of distinguishing between the two ICP phenotypes. In practice, elevated transaminases and pruritus, as common characteristics and symptoms of pregnant women with ICP, are easily measurable and identifiable, allowing for classification and proactive monitoring and management of high-risk ICP pregnancies based on symptom comorbidity. We recommend more aggressive monitoring and treatment strategies for ICP-M patients to reduce the risk of adverse outcomes. However, our current classification is based solely on TBA and related characteristics at the time of diagnosis. Future studies should incorporate repeated measurements and careful monitoring of TBA levels in conjunction with clinical symptoms to provide a more accurate and reliable assessment of the patient’s risk status. This approach would enhance the effectiveness of our classification system and allow for more personalized treatment plans, ultimately improving the efficiency and effectiveness of clinical diagnosis and treatment.

Furthermore, the previous ZEBRA obstetric cohort included only 665 cases of ICP-M, and epidemiological inferences for subgroups within the ICP-M, particularly those with fewer than 50 cases, were not robust enough to warrant subtype classification (19). Based on a larger cohort, this study confirms that the risk of adverse pregnancy outcomes for women with ICP-M_B_ is significantly higher than for those with single-symptom ICP-M_p_ or ICP-M_T_; however, no significant differences were observed in perinatal outcomes between ICP-M_p_ and ICP-M_T_. In particular, the risk of vaginal laceration and perineal laceration in ICP-M_B_ was significantly lower than ICP-M_p_, potentially benefiting from higher levels of monitoring and treatment associated with ICP-M_B_. Elevated transaminase levels may prompt physicians to be more concerned about liver function abnormalities, leading to more aggressive interventions such as more frequent prenatal visits, earlier induction of labor, or cesarean delivery to reduce stress and potential complications during childbirth. Moreover, ICP-M_B_ patients may receive stricter obstetric management, such as the use of more meticulous techniques during labor to control the progress of delivery and reduce the risk of perineal tears. These management measures may help to lower the incidence of physical childbirth injuries, although these hypotheses require further verification through clinical research.

Over the past few decades, UDCA has become the preferred medication for treating ICP (20–22). UDCA improves the pathological state of cholestasis effectively by upregulating metabolic enzymes and bile acid transport proteins in the liver, thus enhancing the excretion of bile acids (23). Although UDCA has shown significant efficacy in improving clinical symptoms of ICP patients, particularly pruritus, with several studies demonstrating its benefits in alleviating itchiness, its effect remains controversial. For example, the PITCHES trial did not find a significant benefit of UDCA in reducing pruritus and adverse perinatal outcomes (11, 24). The symptomatic improvement with UDCA may indicate varying impacts on clinically classified ICP subtypes; hence, this study analyzes the effects of UDCA and second-line medication SAMe on adverse perinatal outcomes across different clinical subtypes of ICP. However, the use of medication did not rescue the adverse pregnancy outcomes in various ICP subtypes. In particular, given the higher risk of preterm birth in patients with ICP-M and those treated with second-line medication, and no significant difference in risk with UDCA use across subtypes, this suggests that UDCA may be more effective than SAMe in preventing preterm birth in the high-risk ICP-M subtype.

Guidelines for the timing of delivery are crucial for optimally balancing the risks of morbidity associated with early or late deliveries. By employing the RCS to analyze the nadir of NICU admission, this study found that in both the ICP-M and ICP-S groups, the risk of NICU admission tends to decline as the timing of delivery shifts from late preterm to full term. This suggests that opting for full-term delivery has a positive impact on reducing the risk of severe neonatal adverse outcomes, regardless of the ICP clinical subtype. ICP-S pregnancies may consider delivery upon reaching full term; for high-risk ICP-M pregnancies, particular attention should be paid to the low-risk delivery window between 38 and 40 weeks to avoid increased risks of adverse neonatal outcomes due to delayed delivery. By selecting late preterm and full-term ICP cohorts, this study expands the exploration of the risk of neonatal morbidity associated with the timing of delivery, providing evidence-based recommendations for singleton pregnancies across different ICP subtypes. However, it is important to note that the number of participants who delivered after 40 weeks was very small in both the ICP-S (two participants) and ICP-M (seven participants) groups. This limitation in sample size may affect the strength of our conclusions regarding the optimal timing of delivery in these subtypes, especially for term deliveries. Future studies with larger sample sizes are needed to further validate our findings in this specific time frame.

### Limitations

In this study, we investigated the impact of different clinical subtypes of ICP on perinatal outcomes and how to determine the optimal timing of delivery to reduce the risk of NICU admission. However, as a retrospective cohort study, we faced several limitations. First, due to the nature of retrospective research, we were unable to obtain and quantify potential confounding factors such as dietary habits, physical activity levels, and mental health status of pregnant women with ICP. These factors could all influence pregnancy outcomes, and our inability to consider these variables in our data analysis may limit our comprehensive understanding of the different impacts of ICP clinical subtypes. At the same time, GDM is typically diagnosed between 24 and 28 weeks of gestation, whereas ICP may occur during the second or third trimester of pregnancy. The treatment for GDM, such as dietary management and insulin therapy, may influence the risk for ICP. This temporal difference introduces uncertainty in causal relationships when evaluating certain outcomes, possibly due to the retrospective nature of the studies. Second, the study lacked detailed information regarding the delivery intentions of pregnant women, the indications for delivery, and whether prenatal fetal monitoring was conducted. These factors are particularly important as they may influence the timing of delivery and subsequent neonatal outcomes. For instance, in cases involving ICP, the decision to deliver prematurely may reflect specific medical considerations aimed at safeguarding the health of both the mother and the child. The distinction between elective deliveries and medically indicated deliveries is crucial for understanding how the timing of delivery impacts birth weight and other neonatal outcomes. The lack of this information may limit our ability to fully elucidate the relationship between the timing of delivery and neonatal outcomes. In addition, it is noteworthy that the home isolation during the SARS-CoV-2 pandemic may have further impacted the vulnerable ICP women. Previous studies have shown that home isolation exacerbated health issues in women with ICP, leading to significantly higher bile acid levels in the isolated group, as well as a higher incidence of adverse pregnancy outcomes, such as GDM, preeclampsia, postpartum hemorrhage, and placental implantation. These factors may further affect the accuracy of our study conclusions. Finally, as this study utilizes a dual-center ICP specialty cohort database, future research could match a certain number of non-ICP populations for validation and further investigation. In addition, early preterm birth is a subgroup worthy of in-depth study, especially in the exploration of the optimal gestational week for delivery. Therefore, future research with larger sample sizes is needed to specifically investigate early preterm birth, in order to fill the research gap in this area.

## Data Availability

The raw data supporting the conclusions of this article will be made available by the authors, without undue reservation.
