# Supplementary material for: Investigating the risks of late preterm and term neonatal morbidity across clinical subtypes of intrahepatic cholestasis of pregnancy
Source: Front Med (Lausanne). 2025 Mar 14;12:1528705. doi: 10.3389/fmed.2025.1528705 (PMC11949799; doi:10.3389/fmed.2025.1528705)
Supplement: Supplementary file 1 [file Supplementary_file_1.docx]

**Investigating the Risks of Late Preterm and Term Neonatal Morbidity Across Clinical Subtypes of Intrahepatic Cholestasis of Pregnancy**

Wei-Zhen Tang ^a,b^, Yi-Fan Zhao ^a^, Lan Wang ^b^, Qin-Yu Cai ^a,b^, Wei-Ze Xu ^a^, Li Wen ^b^, Xue-Bing Chen ^a^, Ting-He Sheng ^a,c^, Tian-Qi Fan ^a^, Tai-Hang Liu ^a,^*, Rong Li ^c,^*, Shang-Jing Liu ^a,^*

**Affiliations:**

^a^ Department of Bioinformatics, School of Basic Medical Sciences, Chongqing Medical University, Chongqing 400016, China.

^b^ Department of Obstetrics and Gynecology, Women and Children’s Hospital of Chongqing Medical University, Chongqing 401147, China.

^c^ Department of Obstetrics, The First Affiliated Hospital of Chongqing Medical University, Chongqing 400016, China

* **Correspondence:**

Tai-Hang Liu [(liuth@cqmu.edu.cn);](mailto:(liuth@cqmu.edu.cn);) Rong Li [(cqums.lirong@163.com);](mailto:(cqums.lirong@163.com);) Shang-Jing Liu (liushangjing@cqmu.edu.cn).

Box 197, Chongqing Medical University, No.1 Yixueyuan Rd, Chongqing, 400016, PR China. Tel.: +86 023 68485868.

**Supplementary Table 1. ICP-M is associated with an increased risk of maternal and neonatal outcomes compared to ICP-S.**

|  | **Model1** |  | **Model2** |  |
| --- | --- | --- | --- | --- |
| **Outcomes** | **cOR (95%CI)** | ***P*-value** | **aOR (95%CI)** | ***P*-value** |
| **Maternal outcome** |  |  |  |  |
| Gestational diabetes | 1.61(1.26,2.05) | <0.001* | 1.57(1.23,2.01) | <0.001* |
| Gestational hypertension | 1.75(0.91,3.37) | 0.094 | 1.64(0.88,3.33) | 0.143 |
| Preeclampsia | 0.94(0.61,1.43) | 0.755 | 0.85(0.56,1.32) | 0.454 |
| Cervical laceration | 1.22(0.64,2.33) | 0.550 | 1.25(0.67,2.50) | 0.501 |
| Vaginal laceration | 0.98(0.71,1.36) | 0.910 | 1.01(0.73,1.41) | 0.961 |
| Laceration perineum | 0.84(0.61,1.15) | 0.271 | 0.87(0.63,1.20) | 0.372 |
| **Neonatal outcome** |  |  |  |  |
| Premature delivery | 2.01(1.47,2.74) | <0.001* | 1.92(1.41,2.67) | <0.001* |
| Low birth weight infant | 1.81(1.30,2.52) | <0.001* | 1.76(1.26,2.51) | 0.001* |
| Hyperamniotic fluid | 2.16(0.97,4.83) | 0.060 | 2.05(0.97,5.02) | 0.082 |
| Macrosomia | 0.67(0.38,1.19) | 0.170 | 0.66(0.37,1.20) | 0.156 |
| FGR | 1.46(0.64,3.34) | 0.372 | 1.39(0.64,3.48) | 0.434 |
| Fetal distress | 0.90(0.63,1.29) | 0.572 | 0.92(0.65,1.32) | 0.634 |
| NICU admission | 1.58(1.07,2.32) | 0.020* | 1.48(1.01,2.22) | 0.046* |
| Amniotic fluid stool staining | 0.99(0.76,1.30) | 0.964 | 1.00(0.77,1.32) | 0.982 |

**Abbreviations:** ICP, Intrahepatic cholestasis of pregnancy; ICP-S, Single-symptom ICP; ICP-M, Multi-symptomatic ICP; NICU admission, Neonatal intensive care unit admission

*P*-values were calculated using logistic regression analysis. Crude odds ratios (cOR) and adjusted odds ratios (aOR) were estimated with 95% confidence intervals (CI). Differences marked with * indicate significance at *P* < 0.05.

**Supplementary Table 2. The pre-delivery outcomes and characteristics of ICP patients with different clinical subtypes.**

|  | **ICP-S group (n =520)** | **ICP-M group (n =1,537)** | ***P*-value** |
| --- | --- | --- | --- |
| TBA during childbirth | 12.90[9.50,21.30] | 14.80[10.10,26.30] | <0.001* |
| Cinical subtypes during childbirth |  |  | <0.001* |
| Classified as ICP-S | 496(95.39) | 533(34.68) |  |
| Classified as ICP-M | 24(4.62) | 1004(65.32) |  |

**Abbreviations:** ICP, Intrahepatic cholestasis of pregnancy; ICP-S, Single-symptom ICP; ICP-M, Multi-symptomatic ICP

*P*-values were calculated using the Mann-Whitney U test for continuous variables presented as median [IQR] and the chi-square test for categorical variables presented as n (%). Differences marked with * indicate significance at *P* < 0.05.

**Supplementary Table 3. The pre-delivery outcomes and characteristics of ICP patients with different clinical subtypes.**

| Outcomes | ICP-M_T_ (n = 861) | ICP-M_P_ (n =156) | ICP-M_B_ (n =520) | P-value |
| --- | --- | --- | --- | --- |
| TBA during childbirth | 15.10[10.40,25.40] | 11.70[7.30,23.80] | 15.30[10.10,28.20] | <0.001* |
| Cinical subtypes during childbirth |  |  |  | <0.001* |
| Classified as ICP-S | 346(40.19) | 63(40.39) | 124(23.85) |  |
| Classified as ICP-M | 515(59.81) | 93(59.62) | 396(76.15) |  |

**Abbreviations:** ICP, Intrahepatic cholestasis of pregnancy; ICP-MT, high [sTBA](https://www.sciencedirect.com/topics/biochemistry-genetics-and-molecular-biology/bile-acid) with only high [transaminase](https://www.sciencedirect.com/topics/medicine-and-dentistry/transaminase) levels ICP; ICP-MP, high sTBA with only pruritus ICP; ICP-MB, high sTBA with both high [transaminase](https://www.sciencedirect.com/topics/pharmacology-toxicology-and-pharmaceutical-science/aminotransferase) levels and pruritus ICP; Intrahepatic cholestasis of pregnancy; ICP-S, Single-symptom ICP; ICP-M, Multi-symptomatic ICP

*P*-values were calculated using the Kruskal-Wallis test for continuous variables presented as median [IQR] and the chi-square test for categorical variables presented as n (%). Differences marked with * indicate significance at *P* < 0.05.

**Supplementary Table 4. The impact of UDCA and the second-line drug SAMe on patients with different clinical subtypes of ICP at the time of diagnosis.**

|  | **ICP-S** |  |  |  |  |  | **ICP-M** |  |  |  |  |  |
| --- | --- | --- | --- | --- | --- | --- | --- | --- | --- | --- | --- | --- |
| **Outcomes** | **UDCA** |  | ***P*-value** | **SAMe** |  | ***P*-value** | **UDCA** |  | ***P*-value** | **SAMe** |  | ***P*-value** |
|  | **Take** | **Not take** |  | **Take** | **Not take** |  | **Take** | **Not take** |  | **Take** | **Not take** |  |
| TBA during childbirth | 12.00[7.50,23.90] | 13.20[10.40,20.70] | 0.069 | 10.20[7.00,17.60] | 12.90[9.60,21.40] | 0.452 | 13.80[8.60,25.40] | 15.80[10.70,26.80] | <0.001 | 14.40[9.00,25.30] | 14.90[10.10,26.30] | 0.546 |
| Cinical subtypes during childbirth |  |  | 0.174 |  |  | 0.592 |  |  | <0.001 |  |  | 0.401 |
| Classified as ICP-S | 170(97.14) | 326(94.49) |  | 12(92.31) | 484(95.46) |  | 254(43.12) | 279(29.43) |  | 44(38.26) | 489(34.388) |  |
| Classified as ICP-M | 5(2.86) | 19(5.51) |  | 1(7.69) | 23(4.54) |  | 335(56.88) | 669(70.57) |  | 71(61.74) | 933(65.61) |  |

**Abbreviations:** ICP, Intrahepatic cholestasis of pregnancy; ICP-S, Single-symptom ICP; ICP-M, Multi-symptomatic ICP; UDCA, Ursodeoxycholic Acid; SAMe, S-adenosylmethionine

*P*-values were calculated using the Mann-Whitney U test for continuous variables presented as median [IQR] and the chi-square test for categorical variables presented as n (%). Differences marked with * indicate significance at *P* < 0.05.

**Supplementary Table 5**. Incidence of maternal and neonatal morbidity postpartum, stratified by gestational week according to different clinical subtypes of ICP.

| **Morbidity** | **Week Gestation** | **ICP-S group** | | **ICP-M group** | | ***p*-value** |
| --- | --- | --- | --- | --- | --- | --- |
|  |  | **Neonates Delivered (N)** | **Number with Outcome (%)** | **Neonates Delivered (N)** | **Number with Outcome (%)** |  |
| NICU admission | 34^0^–34^6^ | 6 | 30.00 | 36 | 38.71 | 0.465 |
|  | 35^0^–35^6^ | 4 | 17.39 | 48 | 39.02 | 0.047* |
|  | 36^0^–36^6^ | 12 | 27.91 | 30 | 16.95 | 0.101 |
|  | 37^0^–37^6^ | 1 | 1.09 | 17 | 5.94 | 0.057 |
|  | 38^0^–38^6^ | 6 | 4.35 | 7 | 1.72 | 0.080 |
|  | 39^0^–39^6^ | 3 | 2.29 | 8 | 2.51 | 0.892 |
|  | 40^+^ | 2 | 2.74 | 7 | 5.34 | 0.385 |

Abbreviations: ICP, Intrahepatic cholestasis of pregnancy; ICP-S, Single-symptom ICP; ICP-M, Multi-symptomatic ICP; NICU admission, Neonatal intensive care unit admission

*P*-values were calculated using the chi-square test for categorical variables presented as number with outcome (%). Differences marked with * indicate significance at *P* < 0.05.

**Supplementral Table 6**. Association between gestational age at delivery and NICU outcomes across different clinical subtypes of ICP.

|  | **Total** | | | | **ICP- S group** | | | | **ICP - M group** | | | |
| --- | --- | --- | --- | --- | --- | --- | --- | --- | --- | --- | --- | --- |
|  | **Model1** | | **Model2** | | **Model1** | | **Model2** | | **Model1** | | **Model2** | |
| Week of Gestation | cOR (95%CI) | *p*-value | aOR (95%CI) | *p*-value | cOR (95%CI) | *p*-value | aOR (95%CI) | *p*-value | cOR (95%CI) | *p*-value | aOR (95%CI) | *p*-value |
| 34^0^–34^6^ | 11.83(6.54,22.19) | <0.001* | 10.91(5.99,20.58) | <0.001* | 39.00(6.07,766.55) | 0.001* | 39.89(6.08,791.20) | 0.001* | 9.99(5.33,19.43) | <0.001* | 9.00(4.75,17.64) | <0.001* |
| 35^0^–35^6^ | 11.06(6.29,20.27) | <0.001* | 10.58(5.99,19.47) | <0.001* | 19.16(2.66,386.22) | 0.010* | 19.80(2.69,403.22) | 0.010* | 10.13(5.61,19.09) | <0.001* | 9.53(5.25,18.05) | <0.001* |
| 36^0^–36^6^ | 4.72(2.68,8.62) | <0.001* | 4.54(2.57,8.31) | <0.001* | 35.23(6.55,654.87) | 0.001* | 33.97(6.24,634.22) | 0.001* | 3.23(1.74,6.17) | <0.001* | 3.10(1.67,5.94) | <0.001* |
| 37^0^–37^6^ | 1.00 (Reference) |  | 1.00 (Reference) |  | 1.00 (Reference) |  | 1.00 (Reference) |  | 1.00 (Reference) |  | 1.00 (Reference) |  |
| 38^0^–38^6^ | 0.49(0.23,1.01) | 0.053 | 0.53(0.25,1.08) | 0.084 | 4.14(0.69,78.82) | 0.192 | 4.27(0.70,82.06) | 0.186 | 0.28(0.11,0.65) | 0.005* | 0.30(0.11,0.70) | 0.008* |
| 39^0^–39^6^ | 0.50(0.23,1.06) | 0.076 | 0.54(0.25,1.15) | 0.118 | 2.13(0.27,43.49) | 0.515 | 2.31(0.29,47.39) | 0.475 | 0.41(0.16,0.93) | 0.040* | 0.43(0.17,0.99) | 0.047* |
| 40^+^ | 0.92(0.39,2.04) | 0.848 | 1.001(0.42,2.23) | 0.991 | 2.56(0.24,55.81) | 0.446 | 2.70(0.25,59.14) | 0.425 | 0.89(0.34,2.13) | 0.807 | 0.97(0.37,2.31) | 0.941 |

Abbreviations: ICP, Intrahepatic cholestasis of pregnancy; ICP-S, Single-symptom ICP; ICP-M, Multi-symptomatic ICP

Mode 1: cOR values were unadjusted; Mode 2: aOR values were adjusted for Maternal age、Pre-gestation BMI、Nullipara, Weeks of diagnosis and IVF.

**p* <0.05
